# Report of a Case of Acute Laryngitis, in Which Laryngo-Tracheotomy Was Performed

**Published:** 1864-12

**Authors:** Ignatius D. Thompson

**Affiliations:** Assistant-Surgeon P. A. C. S.


ART. III.—*Report of a Case of Acute Laryngitis, in which Laryngo-Tracheotomy was performed.* By IGNATIUS D. THOMPSON, Assistant-Surgeon P. A. C. S.

About midnight, on the 14th of March, 1863, was called to Private Davis, in General Hospital No. 2, Lynchburg, Va. Discovered the case to be one of acute laryngitis, with œdema of the glottis; patient leaning anxiously forward, laboring for breath; countenance distorted, throat and neck much swollen; could speak only in whispers and with much difficulty; pulse weak and irregular, and skin bathed in clammy sweat.

Private Davis had been admitted into hospital ten days previously, with chronic rheumatism, and, until this night, had been entirely free from any disease of respiratory organs. On going to bed, about eight o'clock that evening, had complained of sore throat, but was not considered ill. A little before midnight he aroused the nurse and said he was dying of suffocation.

Emollient poultices and derivations, together with antimony, in nauseating doses, gave temporary relief; but in a few hours the worst symptoms returned, and soon the power of respiration was lost entirely. The patient, completely asphyxiated, struggled violently for a short time, and then, to all appearances, was dead. A thumb-lancet was the only surgical instrument at hand; with it, laryngo-tracheotomy was immediately performed. The incision was made in the median line, through the crico-thyroid membrane, cricoid cartilage and first ring of the trachea.

There was much less hemorrhage than, from the vascularity of the part and asphyxiated condition of patient, might have been anticipated.

Artificial respiration was now practised for nearly two minutes, when a little blood and some frothy mucus were expelled through the opening, and the lungs resumed their functions.

The patient soon recovered consciousness, and presently expressed himself, in writing, as greatly surprised at finding that he was "alive again."

The handle of a thumb-lancet, turned transversely in the opening, afforded sufficient air, and, after daylight, one of Obre's admirably constructed trachea tubes was obtained and carefully fitted into the opening. Nourishment and stimulants were now administered through a gum catheter which was passed into the œsophagus.

My subsequent knowledge of the case is due to the courtesy of the medical officer in charge of the ward, (in whose temporary absence I had been called upon.)

On the second day after the operation the swelling of the throat had subsided and the patient so far recovered as to be able to leave his bed and could take fiuids in the natural way.

During the second night after the operation, either from accidental displacement of the tube or the clogging of it with mucus, the patient suddenly expired.

A *post mortem* examination proved the correctness of the diagnosis.

It is believed that this should be regarded as a successful operation, as the death of the patient resulted from an accident which might always be averted.

